# A Comparison of the Mechanical Properties of ECM Components and Synthetic Self‐Assembling Peptides

**DOI:** 10.1002/adhm.202402385

**Published:** 2025-02-19

**Authors:** Alex Hartley, Philip Michael Williams, Alvaro Mata

**Affiliations:** ^1^ School of Pharmacy, University of Nottingham University Park Campus Nottingham NG7 2RD UK; ^2^ Biodiscovery Institute, University of Nottingham University Park Campus Nottingham NG7 2RD UK; ^3^ Department of Chemical and Environmental Engineering, University of Nottingham University Park Campus Nottingham NG7 2RD UK

**Keywords:** breaking strain, collagen I, elastin, fibronectin, Fmoc‐FF, loss modulus, MAX1, RADA16‐I, storage modulus, tangent modulus, viscoelasticity, Young's modulus

## Abstract

The field of tissue engineering is increasingly moving away from a one‐size‐fits‐all approach of simple synthetic homogeneous gels, and embracing more tailored designs to optimize cell function and differentiation for the organ of interest. Extracellular matrix (ECM) proteins are still the optimal route for controlling cell function, while a field of great promise is that of synthetic self‐assembling peptides (SSAPs), which are fully biocompatible, biodegradable, and offer both the hierarchical structure and dynamic properties displayed by protein networks found in natural tissue. However, the mechanical properties of neither group have been comprehensively reviewed. In this review, rheological data and the Young's modulus of the most prevalent proteins involved in the ECM (collagen I, elastin, and fibronectin) are collated for the first time, and compared against the most widely researched SSAPs: peptide amphiphiles (PAs), β‐sheets, β‐hairpin peptides, and Fmoc‐based gels (with a focus on PA‐E3, RADA16, MAX1, and FmocFF, respectively).

## Graphical abstract

Self‐assembling peptide hydrogels are increasingly utilized in biomedical applications. The authors review the key mechanical properties of these structures across multiple size scales and compare them to that of native extracellular matrix components, which are found to display a much wider range of structural attributes. The authors then consider these findings in relation to cell response and future biomaterial design.

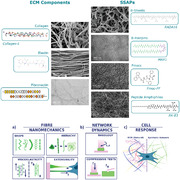

## 1 Introduction

The extracellular matrix (ECM) is a complex hydrated milieu that regulates cell behavior. Insoluble ECM components are excreted by cells and assemble into diverse amorphous and fibrillar organizations that protect cells, enable communication, regulate cell migration, and facilitate storage of signaling molecules. By binding to these structures, cells are able to mechanically sense their surroundings over distances of tens of micrometers,^[^ ^1^ ^]^ which is critical for their survival and cooperative functionalities such as where to migrate, when to differentiate, and when to undergo apoptosis.^[^ ^2^, ^3^ ^]^ Furthermore, the high porosity generated by this network ensures that all pockets of fluid within it are linked, allowing nutrients and waste products to diffuse,^[^ ^4^ ^]^ facilitating functionality, communication and survival.

The insoluble fibril network component of the ECM collectively acts as a single nanofibrous material, producing a wide variety of bioactive and dynamic environments for different cells. However, this material is composed of different types of key nanofibers exhibiting distinct chemical, mechanical and biological properties. The main types of nanofibers are proteinous in nature and include collagen I, elastin, and fibronectin. Collagen I is a stiff structural protein present in most tissues. Elastin is an entropically elastic protein with a critical role in the skin, lungs and tendons. Fibronectin acts as a glue to bind ECM components together, containing many growth factor binding sites and predominating during fetal development and wound healing. In addition, the ECM also comprises an amorphous component comprised of proteoglycans and glycosaminoglycans such as hyaluronic acid, whose role in reducing shear‐stress and damping are crucial for tissues such as the eye, muscles, and in synovial fluid.^[^ ^5^ ^]^

The clear distinctions in mechanical properties between the various bodily tissues are produced via compositional differences in the proportions of each component of the ECM.^[^ ^2^ ^]^ The intrinsic heterogeneity of the ECM enables the potential production of an almost infinite number of combinatorial states using only a finite number of protein and glycan building blocks, allowing finite control over growth‐factor levels, diffusion length, and ligand availability. In this way, nature has evolved mechanisms to optimize tissue properties such as bulk moduli, stiffness, elasticity, energy storage and hydration. Understanding the mechanical properties of individual ECM components is therefore critical to recreating both the structure and function of tissues and has implications in areas such as regenerative medicine, disease modeling, and cultivated meat.

In an effort to recreate the critical functionality of the ECM, the last twenty years have seen an increasing interest in synthetic fibrous materials based on molecular building blocks such as peptides^[^ ^6^, ^7^ ^]^ and polymers.^[^ ^8^, ^9^, ^10^ ^]^ In particular, synthetic self‐assembling peptide hydrogels (SSAPHs) have been more recently gaining interest due to their precise structure and biocompatibility.^[^ ^6^, ^11^, ^12^, ^13^, ^14^, ^15^ ^]^ SSAPs can be produced in highly pure concentrations via solid‐phase peptide synthesis^[^ ^16^ ^]^ and assemble via non‐covalent interactions such as hydrogen bonds and hydrophobic forces into well‐defined nanofibers capable of recreating functional and architectural features of the natural ECM.^[^ ^17^ ^]^ Furthermore, the modular nature of these synthetic matrices also offer the capacity to engineer properties such as bioactivity,^[^ ^17^ ^]^ adaptability,^[^ ^18^ ^]^ and tunability of mechanical properties,^[^ ^19^, ^20^, ^21^ ^]^ degradation,^[^ ^22^ ^]^ and signaling.^[^ ^23^ ^]^ These functionalities have enabled the design of synthetic matrices capable of recreating features of in‐vivo environments in vitro such as bone spheroids,^[^ ^24^ ^]^ brain organoids,^[^ ^25^ ^]^ and tumor microenvironments.^[^ ^26^, ^27^ ^]^

**Figure 1:**
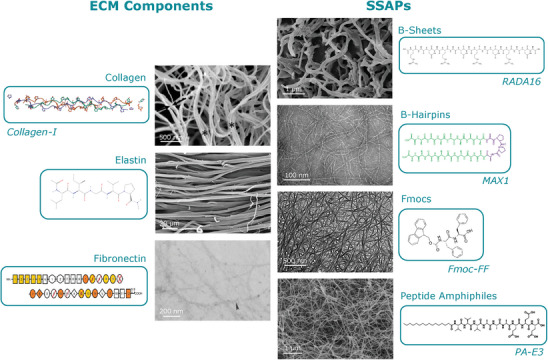
Visualizations of ECM components and SSAP fibers. Monomer diagrams of specific fiber subgroups are displayed as inserts, alongside AFM or SEM images of their fibril networks. Collagen‐I insert reproduced from the 3B2C entry of RCSB PBD.^[^ ^34^, ^35^ ^]^ Collagen image: adapted with permission.^[^ ^36^ ^]^ 2012, Elsevier. Elastin insert reproduced from the PubChem archive.^[^ ^37^ ^]^ Elastin image: adapted with permission.^[^ ^38^ ^]^ 2007, Elsevier. Fibronectin insert: reproduced under the terms of the CC‐BY license.^[^ ^39^ ^]^ 2021, MDPI. Fibronectin image: adapted with permission.^[^ ^40^ ^]^ 1966, Company Of Biologists. RADA16 insert: adapted under the terms of the CC‐BY license.^[^ ^41^ ^]^ 2022, MDPI. RADA16 image: reproduced under the terms of the CC‐BY license^[^ ^42^ ^]^ 2023, MDPI. MAX1 insert: adapted under the terms of the CC‐BY license^[^ ^43^ ^]^ 2025, PNAS. MAX1 image: reproduced under the terms of the CC‐BY license^[^ ^44^ ^]^ 2021, Frontiers. Fmoc‐FF insert and image: reproduced/adapted under the terms of the CC‐BY license^[^ ^45^ ^]^ 2014, PNAS. PA‐E3 insert and image: reproduced/adapted with permission.^[^ ^46^ ^]^ 2010, ACS Publications.

While the mechanical properties of tissues and organs are well established,^[^ ^28^, ^29^ ^]^ there is little known about the properties of their constituent components. Similarly, while the calculation of the storage modulus of a new SSAP gel is common practice in the literature,^[^ ^30^, ^31^, ^32^, ^33^ ^]^ there is limited information on the mechanical properties of individual SSAP nanostructures. The common properties of SSAPs and fibrous ECM components (i.e., that both are proteinous, fibrous, and compile via self‐assembly) uniquely enable the direct comparison of these structures over multiple size scales: from assembly characteristics, to fiber organization, to hydrogel structure. In this review article, instead of comparing the properties of SSAPHs to specific tissues, we dissect the fibrous component of the ECM into its constituent proteinous nanofibers to compile their individual mechanical properties, and directly compare them to the mechanical properties of SSAPs. We first introduce fundamental concepts of mechanical properties that are relevant to the ECM. Then, we describe the hierarchical structure and mechanical properties of four critical ECM proteins including collagen‐I, elastin, and fibronectin, as well as four broadly‐used SSAPs including MAX1, RADA16, Fmoc‐FF, and PA‐E3 (**Figure** **1**). We discuss the effect of their amino acid sequences on their structural properties and compare their viscosity maps, fiber diameters, breaking strains and Young's moduli. Finally, in an effort to shed light on the role of individual molecular building‐blocks on the properties of ECMs and tissue, we discuss the link between the physio‐chemical properties of these molecules to their effects on cell mechanotransduction.

## 2 Hierarchical Mechanics

In SSAPs, macroscale mechanical properties are fundamentally determined by nanoscale interactions. This bottom‐up, hierarchical approach to biomaterial production enables them to produce complex and functional structures with only simple building blocks. While the complexity of the monomers of fibrous ECM components is far greater than that of any SSAP, the underlying process of self‐assembly into complex functional networks is highly similar. Such structures interact dynamically at the nanoscale to produce stable macrostates, and possess different mechanical properties dependent on the measured scale. In order to quantitatively compare these mechanical properties across multiple materials, it is critical that a measurement technique must satisfy two criteria: first, the technique must be comparable across material type. This means that the method should provide consistent and meaningful results whether it is applied to soft or hard materials, over different pH and temperatures, ensuring that measurements can be reliably compared between different materials. Second, the measurement technique must be comparable across multiple size scales. This criterion ensures that the technique remains valid and produces consistent results whether it is used to measure properties at the nanoscale, microscale, or macroscale, allowing for comprehensive analysis across various dimensions of a material or system. To this end we have focused on collating measurements exploring the stiffness, viscoelasticity, and breaking strain of the materials in this review, which can be measured over the multiple size scales explored in this study and allow us to quantitatively compare the mechanical properties of ECM components and SSAPHs. Such measurement techniques include rheometry, AFM nanoindentation, and tensile and compressive testing, that enable comparison between materials. Furthermore, we have focused on developing comparison while taking into account the size scale at which each technique operates. In this way, we are able to quantitatively compare the mechanical properties of ECM components and SSAPHs. The following section gives detail on these mechanical concepts.

### 2.1 Nanoscale Mechanics

**Figure 2:**
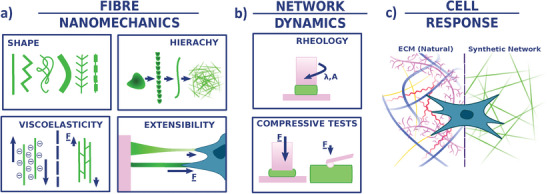
a) SSAPs represent complex chemical and mechanical environments whose many microstates interact en masse to produce a predictable macrostate structure over several size scales. Slight changes in monomer composition will significantly alter the material's macrostates by changing both monomer and fiber interactions, and therefore the nanomechanics of the system. b) Interfiber interactions result in network dynamics which can be measured via techniques such as rheology and AFM. c) Cell‐matrix interactions lie between the nano‐ and micro‐scale, where the cell binds to its surroundings at the monomer level via, e.g., integrins,^[^ ^50^ ^]^ but is also able to respond to an environment tens of micrometers away.^[^ ^1^ ^]^ The exact contributions of fiber mechanics vs network mechanics to cell response are not well understood, and differs greatly between natural ECM and synthetic hydrogels. Illustration is the author's own work.

Organic molecules are typically formed of predominately carbon, nitrogen, oxygen, and hydrogen atoms which naturally favor the formation of strong elastic covalent bonds. Proteins form when amino acids bind together via peptide bonds, resulting in a repeating NCCNCC backbone with bond energies of 205 kJ mol^−1^ for the C–N bond, and 346 kJ mol^−1^ for the C‐C bond.^[^ ^47^ ^]^ Methionine and Cysteine amino acids also contain sulfur groups, which can bind together with a smaller bond energy of 14.5 kJ mol^−1^.^[^ ^48^ ^]^ Another type of interaction is hydrogen bonds, which are electrostatic interactions between a hydrogen atom and an electronegative atom. These interactions are an intrinsic part of aqueous media. Hydrogen bonds are much weaker than other bond types with a range of only 8–62 kJ mol^−1^,^[^ ^49^ ^]^ but they are vital to the folding and association of peptides to define the secondary and tertiary structure of proteins, and therefore their mechanical properties. Buehler and Ketan (2008) found that three common protein secondary structures showed considerable variation in breaking strain and elastic modulus. For example, tropocollagen gradually stiffened in response to an increase in strain until the molecule broke apart, while alpha helices initially softened in response to increasing strain but later experienced a second stiffening event under high strains. In contrast, β‐sheets exhibited consistently high stiffness under low strains until a cutoff point, where greater strain triggered a sudden catastrophic rupture. Here, we start to see how elastic interactions at the atomic scale are able to produce complex emergent mechanics at larger size scales, with measurable differences in protein extension, stiffness, and elasticity profiles. In **Figure** **2**, we seek to explore the relationship between these multi‐scale mechanics and the cell response.

### 2.2 Stiffness

The mechanical properties of soft materials can be described in terms of their stiffness. Simply, the stiffness of a material is the force required to extend that material to a set length. This is often described using the Young's modulus, which can be found by taking the gradient of the linear region of a stress‐strain graph, where “stress” is the force exerted on a material over a set cross‐sectional area, and “strain” is the change in length of an object from its original position. The Young's modulus inherently describes the elastic behavior of the material, as within this linear region the material immediately returns to its original conformation once the force applied to stretch the material is released.^[^ ^51^ ^]^ It is important to note that in a fibrous network such as a hydrogel, the applied force must be shared between the fibrous component and the surrounding fluid, resulting in a reduction in apparent material stiffness.

### 2.3 Viscoelasticity

The viscosity of a fluid is a measure of how easily it flows. This process relies on a reorganization of the macromolecular structure of a fluid. In a fibrous network, viscosity typically manifests as fibril/monomer sliding or crosslinker breakage and reformation, which en masse result in deformation. A viscoelastic material is one that displays both viscous and elastic properties over different forces and timescales. For example, a material may respond elastically to fast strain forces, but viscously to slow strains.^[^ ^51^ ^]^

### 2.4 Breaking Strain

The breaking strain is the maximum strain a material can withstand, where it is stretched past both its elastic and plastic regions until the weakest bonds holding the material together break. The breaking strain of a network may be greater or lesser than the breaking strain of an individual fiber, dependent on the degree and strength of inter‐ and intra‐fiber cross‐linking as well as the flexibility of the network to align along the strain axis. “Strain hardening” is a response to material extension when fibers in the network bend and/or rotate to align with the strain axis, resulting in a period of elastic extension. Stiffening then occurs when all the fibers have bent or aligned to their maximum extent so that further extension can only be produced by stretching of the individual fibers, which requires greater force to achieve.^[^ ^52^ ^]^ At a crucial network density, there are too many fibers for bending/rotation to occur and instead the network plastically deforms, known as “strain softening.”^[^ ^52^, ^53^ ^]^

## 3 ECM Components

The interstitial ECM is a 3D network primarily associated with the mesoderm germ layer which connects basement membranes together. It is primarily composed of collagens, elastin, and fibronectin proteinous macromolecules. The structure of these individual components and their interactions play a key role in the mechanical properties of the ECM.

### 3.1 Collagen I

Collagens are the most abundant proteins in the body.^[^ ^54^, ^55^ ^]^ Collagen I is the most common,^[^ ^52^ ^]^ and is typically found in tissues involved in structural support, such as bone, ligaments, tendons, skin, and vasculature. The collagen monomer is composed of three left‐hand helices which interlock via hydrogen bonds between every third amino acid, glycine, into a stable right‐handed triple‐α‐helix conformation known as tropocollagen.^[^ ^56^, ^57^ ^]^ The monomers combine into parallel overlapping fibrils around 1µm in diameter via a self‐assembling process driven by surface water loss.^[^ ^57^, ^58^ ^]^ Further hydrophobic and polar interactions result in the fibrils combining to produce larger fibers tens of micrometers across,^[^ ^58^ ^]^ which bind together via lysine side chains to produce the strength seen in mature collagen fibers.^[^ ^57^ ^]^

Like most ECM components, collagen is viscoelastic in nature, resulting in a network that stiffens with increased loading force and displays stress relaxation.^[^ ^58^, ^59^ ^]^ This viscoelasticity results from the inherent hierarchical architecture of collagen. At the nanoscale, tropocollagen monomers can be elastically straightened under small, fast‐acting loads, which in combination act like a series of springs. The combined effect is high elastic extension under low strain.^[^ ^60^ ^]^ Precise monomer distribution produces a crimping effect at the microscale, so that under larger, slower forces, these crimped regions straighten out via viscous mechanisms such as inter‐fibril friction. The fibers can remain extended after repeated loading.^[^ ^60^ ^]^ Collagen I networks also strain‐harden at biological concentrations, and strain‐soften above 2.5 mg mL^−1^.^[^ ^52^, ^53^ ^]^

### 3.2 Elastin

Elastin fibers are most prominent in tissues experiencing regular cyclic loading,^[^ ^38^ ^]^ such as the skin and lungs. Fibers are composed of two component parts including microfibrils and tropoelastin. Tropoelastin is a short peptide characterized by repeating glycine‐valine‐proline segments,^[^ ^37^ ^]^ which self‐assembles into fibers in an endothermic process known as coacervation. This process is temperature and pH‐dependent^[^ ^61^ ^]^ and takes place sequentially, starting with tropoelastin aggregating into 2–6 µm‐diameter spherules^[^ ^62^ ^]^ that subsequently fuse 10–12 nm diameter fibrils.^[^ ^38^, ^62^ ^]^

The elastic properties of tropoelastin result from its regular hydrophilic crosslinking domains interspersed with regular hydrophobic domains (GVP), which leads to a coiling effect in solution as each hydrophobic segment attempts to turn inwards into the protein while its adjacent hydrophilic domain faces outwards.^[^ ^62^ ^]^ This crumpling produces spring‐like properties upon extension, as this action exposes the hydrophobic segments of the monomer, which rapidly collapse back into the entropically stable coil upon release.^[^ ^63^ ^]^ This allows a single tropoelastin monomer to elastically extend up to 8 times its original length.^[^ ^38^ ^]^ However, the full elastin fiber contains regular lysine side chains which reduce the fiber's extension potential in favor of greater network strength. Annabi et al. (2010) found that forming elastin networks at high pressure (60 bar CO_2_) produced larger pores and greater crosslinking, resulting in an elastic modulus increase of 250%.^[^ ^64^ ^]^ However, under lower strains and 37°C, elastin follows a neo‐Hookean model,^[^ ^65^, ^66^ ^]^ producing a “hyper‐elastic” linear stress‐strain curve.

### 3.3 Fibronectin

Fibronectin is the first ECM component to assemble during wound healing and fetal development.^[^ ^39^ ^]^ It contains binding sites for integrins, fibrin, heparin and collagen,^[^ ^67^ ^]^ connecting these ECM components together into a fully integrated network. It also contains binding sites for a wide range of growth factors and small molecules important for ECM assembly and remodeling, which allows it to act as a reservoir to control their levels in the surrounding tissue.^[^ ^39^ ^]^ Each fibronectin monomer is actually a soluble glycoprotein dimer of two fibronectin molecules, bound via disulfide bonds.^[^ ^39^ ^]^ These fibronectin molecules are comprised of β‐pleated sheets and a 7‐stranded barrel‐like structure that is able to mechanically deform.^[^ ^67^ ^]^ Fibronectin self‐assembly into fibrils is triggered when a fibronectin dimer binds to a cell surface integrin, triggering the cell body to contract. Once the strain force on the monomer reaches 2–5 nN,^[^ ^39^ ^]^ the monomer's hairpin‐like configuration unfolds into a linear form, exposing binding sites that allow additional dimers to attach along the fibril and in turn trigger further unfolding and binding.^[^ ^39^ ^]^

The sensitivity of fibronectin to mechanical deformation combined with its ability to attach to most ECM components makes it an ideal mechanotransduction apparatus. It is also inherently viscoelastic and experiences creep.^[^ ^68^ ^]^ Paradoxically, fibronectin is found naturally in a state of tension, and will snap back to 20–30% of its original length upon breakage,^[^ ^69^ ^]^ suggesting that cells within the ECM must exert a constantly increasing strain upon the fibronectin network not only to trigger fibrogenesis but also as a normal part of cell–ECM interactions.

## 4 Synthetic Self‐Assembling Peptide Hydrogels

### 4.1 β‐Hairpin Peptides

The first SSAP was designed by Vegner et al. in 1995,^[^ ^70^ ^]^ and was followed 7 years later by the β‐hairpin peptide MAX1 in 2002 by Schneider et al.^[^ ^71^ ^]^ It is a 20‐amino acid peptide, consisting of a repeating pattern of valines and lysines interspersed with a turn sequence halfway along. At low temperatures and pH these molecules remain unfolded, but under alkaline conditions^[^ ^72^, ^73^ ^]^ or an increase in temperature,^[^ ^74^ ^]^ the monomer folds into a tight U‐shape, triggering self assembly. Upon folding, β‐hairpin peptides are amphiphilic, as all the hydrophobic valines and the hydrophilic lysines lie on opposing sides along the protein backbone. The most energetically favorable condition is therefore for two hairpin peptides to stack on top of each other so that the hydrophobic elements are protected within the core of the new protein.^[^ ^74^ ^]^ This occurs in opposing directions to reduce steric hindrance from the linker protein.^[^ ^43^ ^]^ The lysines proceed to rigidify, stabilizing the structure.^[^ ^75^ ^]^. Hydrogen bonds between valines result in stable lateral growth either side of this new monolayer, creating highly uniform 3.5 × 2.5 nm fibrils.^[^ ^43^ ^]^ These assemble random branched clusters, with individual fibrils intersecting their neighbors.

β‐hairpin peptides have shown great promise in the area of biomaterials due to their injectable and broad‐spectrum antibacterial properties.^[^ ^76^, ^77^ ^]^ MAX1 shear‐thins by the gel network fracturing into large (>200 nm) sections that act under pressure as a viscous fluid, but that rapidly reconnect once the shear force is removed, resulting in re‐solidification.^[^ ^76^ ^]^ MAX1 gel stiffness is also tunable via the rate of gel formation, with more rapid assembly producing more rigid gels.^[^ ^74^ ^]^

### 4.2 β‐Sheets

β‐sheet based‐peptides are short peptides between 8 and 32 amino acids in length,^[^ ^78^ ^]^ where one side presents only alanines while the other contains alternating positively and negatively charged amino acids.^[^ ^79^ ^]^ These bind via a combination of hydrophobic association and coulombic attraction of charged amino acids to form β sheets. Examples include RADA16‐I, RADA16‐II, KFE8, KLD12, EAK16‐I, and EAK16‐II.^[^ ^79^ ^]^ RADA16‐I is made up of the repeating sequence RADA,^[^ ^80^ ^]^ whose structure was designed to mimic the receptor binding sequence RGD.^[^ ^79^ ^]^ It has been found to rapidly stop bleeding when added to bodily fluids,^[^ ^81^ ^]^ which combined with its shear‐thinning properties has made it one of the few SSAPHs to be used in clinics.^[^ ^82^ ^]^ Known as PuraStat^[^ ^83^ ^]^ in surgical applications, and PuraMatrix^[^ ^79^ ^]^ in life sciences research, this peptide is able to self assemble rapidly in blood serum and create a network with fiber diameters 20 times greater than that of assembly in water.^[^ ^81^ ^]^ In fact all that is required to initiate gelation with this peptide is the addition of cell culture medium,^[^ ^84^ ^]^ making it highly suited to clinical applications.

### 4.3 Fmoc

Novelly, this small self‐assembling molecule utilizes π‐π stacking of molecules to form fibrils. The monomer is a dipeptide functionalized with an aromatic group,^[^ ^45^ ^]^ which via antiparallel π‐π stacking produces an extended twisted β‐sheet, forming a cylindrical structure around 3 nm in diameter with a hollow center at alkaline pH (9–10).^[^ ^85^ ^]^ When placed under neutral pH, these cylindrical fibrils then stack 5‐wide to create a ribbon.^[^ ^85^, ^86^ ^]^

Fmoc‐FF is one of the simplest of the Fmoc group, and is naturally bactericidal due to electrostatic interactions between bacterial membranes and the gel surface.^[^ ^87^ ^]^ Fmoc‐FF fibers have a hydrated Young's modulus of 1.5 MPa, and a surface energy of 0.1 nN m^−1^.^[^ ^88^ ^]^ However, the mechanical properties of an Fmoc gel are highly sensitive to the gelation procedure; the temperature, pH, mixing rate, agitation, and the addition of, for example, growth factors, can all lead to changes in the final storage modulus of the gel by up to four orders of magnitude.^[^ ^88^, ^89^ ^]^ It is believed that most of these differences are caused by differing proportions of bound and unbound Fmoc molecules, as well as the average fiber thickness.

### 4.4 Peptide Amphiphiles

Peptide amphiphiles (PAs) consist of a hydrophobic tail domain, a β‐sheet forming domain, a glycine spacer and an optional addition of a bioactive peptide to boost cell response.^[^ ^90^ ^]^ In solution the tails associate, and β‐sheet interaction between molecules result in the formation of cylindrical micelles.^[^ ^91^ ^]^ Gelation acts to extend the cylindrical micelles into full fibers, and can be triggered via the addition of multivalent metal ions.^[^ ^14^, ^46^ ^]^ These ions associate with charged groups on the hydrophilic component of PA monomers, allowing hydrogen bonding between adjacent β‐sheet components to take place.

The β‐sheet component of the PA molecule is highly important in determining the bulk mechanical properties of the fibrous network.^[^ ^92^ ^]^ Like most SSAPs, significant differences in PA peptide structure are present throughout the literature. PA‐1 (C_16_A_4_G_3_S(P)KGE‐COOH) was the first, formed by Stendahl et al. in 2006.^[^ ^14^ ^]^ PAE3 is another, comprised of a C_16_ hydrophilic chain bound to β‐sheet sequence V_3_A_3_, which is then attached to three glutamic acids (E_3_) that make up the exterior of the filament. Increasing the length of the β‐sheet has been found to increase the bulk gel stiffness and improve cell viability,^[^ ^91^ ^]^ but increases the gelation time.^[^ ^90^ ^]^ Furthermore, the addition of hydrogen bond‐donors to the β‐sheet forming sequence forms more elastic gels.^[^ ^92^ ^]^ The choice of metal‐ion gelator also directly impacts gel stiffness ‐ Stendahl et al. combined PA‐1 with different metal ions and found that when combined with the monovalent ions Na^+^ and K^+^, PA‐1 created only viscous liquids (1 Pa), while with H^+^ and ${\mathrm{Mg}}_{2}^{+}$ ions soft gels of 100 Pa were formed. Stronger gels could be created with divalent ions such as ${\mathrm{Ca}}_{2}^{+}$ (800 Pa), and ${\mathrm{Fe}}_{2}^{+}$ (10,000 Pa),^[^ ^14^ ^]^ with ${\mathrm{Ca}}_{2}^{+}$ gels able to form stronger inter‐ and intra‐fiber crosslinks than H^+^ gels.^[^ ^46^ ^]^

## 5 Comparison of Mechanical Properties

### 5.1 Stiffness

Material stiffness can be directly compared via the Young's moduli of the materials. Although the Young's modulus of tissues is well documented,^[^ ^28^ ^]^ the values for specific ECM components are less well defined. This is predominantly due to difficulties in isolation of the ECM components from natural tissue: fibers are broken up into their constituent monomers via mechanical and enzymatic degradation, which can result in fragmentation and protein alteration.^[^ ^93^ ^]^ Direct extraction from tissue also results in high batch‐to‐batch variability, making repeatable studies difficult. Additionally, measurement techniques such as tensile testing struggle to measure extremely soft gels or short fibers, so more specialized techniques and equipment must be used which potentially represent a barrier to research. **Table** **1** collates the Young's modulus data present in the literature for collagen, elastin, and fibronectin at the level of individual fibers, dry networks, and hydrated hydrogels, alongside the characterization method utilized and the material source.

**Table 1:** ECM Components Young's Modulus.

		**Characterization Method**	**Young's Modulus (MPa)**	**Source**	**Ref**
**Collagen‐I**	Fiber	AFM buckling (dry)	2,000	Rat tail tendon	[94]
		AFM	15	Rat tail tendon	[95]
		AFM (dry)	14–40	Bovine dermis	[96]
		Tensile Test	6,990–18,820	Computational Simulation	[58]
		Tensile Test	77–169	Sea cucumber	[59]
	Network	Tensile Test, modeled	86–96	Rabbit skin	[97]
		Tensile test, modeled	69–225	Sheep tendon	[98]
	Hydrogel	Tensile test	0.00073–0.00487	Rat tail (Powder) (2 mg/mL)	[99]
		DMA	0.00399–0.00447	Porcine skin (1 mg/mL)	[100]
**Elastin**	Fiber	Tensile Test (dry)	1.2	Bovine ligament	[101]
	Network	Uniaxial Compressive Test	1.38	Pig aorta	[65]
		Tensile test, modeled	1.1	Rabbit skin	[97]
	Hydrogel	Uniaxial Compression Test	0.0079–0.0493	Synthesized (100 mg/mL)	[64]
**Fibronectin**	Fiber	AFM (hydrated)	0.11–0.19	Bovine plasma	[102]

There is a clear difference between the modulus of fibers and those of their hydrogels—fiber stiffness lies in the MPa‐GPa range, while gels comprising these fibers have stiffnesses of only kPa‐Pa. This can be understood from the law of mixtures, which states that the Young's modulus of a composite material is between *E*_*c*_ = *fE*_*f*_ + (1 − *f*)*E*_*m*_ and $E_{c}={\left(\frac{f}{E_{f}}+\frac{1-f}{E_{m}}\right)}^{-1}$, where *E*_*f*_ is the modulus of the fiber component, and *E*_*m*_ is the modulus of the surrounding material. Therefore a 1 mg mL^−1^ gel of fibers of 100 MPa in water (approaching 0 Pa) will produce a maximum elastic modulus of 100 kPa (when the fibers are aligned perpendicular to the direction of strain), and a minimum elastic modulus of 0 Pa (when the fibers are aligned parallel to the direction of strain). If the network is unaligned, the gel would be expected to have an elastic modulus of 50 kPa. This model does not take into account more complex interactions such as branching and crosslinking, but highlights the important dynamic changes that occur when considering a fibrous network in different fluid systems. A hydrogel in pure water will have subtly different mechanical characteristics to one in a more viscous fluid such as PBS or glucose, which will affect the system's compression moduli, friction coefficients between fiber and fluid, as well as the surface hydration of fibers and therefore their packing density and stiffness. Equally, removing the fluid component through drying increases the modulus to a value much closer to that of an individual fiber, as seen in the Table 1. values marked “Network”. Unlike the single‐fiber modulus results, the values measured from these dry networks also take into account inter‐fiber crosslinking, fiber alignment and viscous elements such as inter‐fiber friction. However, the loss of fiber surface hydration during the drying process may severely alter the mechanical properties of the material.

Even when measuring individual fiber stiffness, *E* values for collagen I vary considerably in the literature. The values collated in Table 1 produce a mean estimate collagen fiber stiffness of 3.52±6.6 GPa. This large standard deviation is likely due to either sample preparation or material source, as both the hydration and diameter of a fiber may produce significant differences when taking single‐fiber tensile tests. However, even the smallest elastic modulus of collagen I is still 1–2 orders of magnitude above that of elastin (1.2 MPa^[^ ^101^ ^]^), and fibronectin (0.15 MPa average^[^ ^102^ ^]^).

**Figure 3:**
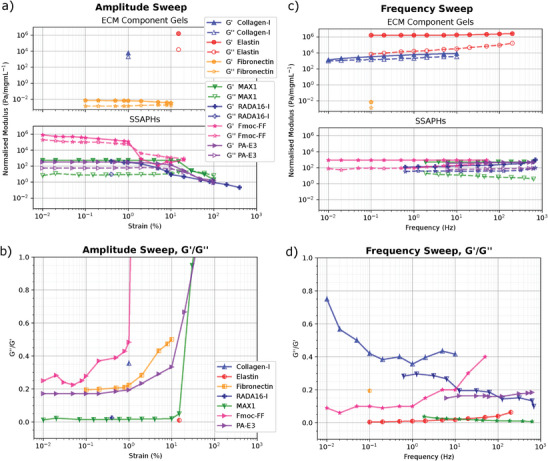
Rheology data collated from the literature, normalized by concentration. For all data only one paper was found per network with full storage and loss moduli data for either amplitude sweep, frequency sweep, or both. Collagen, and elastin had only frequency sweep data, while fibronectin and Fmoc‐FF had only amplitude sweep data. RADA16‐I did not have full loss modulus data throughout its amplitude sweep. Where possible, the parameters from one test have been used to add a data point in the other, with transferred data at either 1% strain or 1 Hz (i.e., a frequency sweep taken at 2% strain has had its data point for 1 Hz shear added to the amplitude sweep graph at 2%). a) Amplitude sweep data. Breaking strain is observed when the storage modulus is no longer linear, and the loss modulus equals or becomes greater than the storage modulus. b) Tangent modulus (G′/G″) of the amplitude sweep data. c) Frequency sweep data. For reference, angular frequency of walking is around 0.1 Hz,^[^ ^104^ ^]^ while a typical heart rate is between 0.7 Hz and 2 Hz. d) Tangent modulus (G′/G″) of the frequency sweep data. **References and parameters**: Collagen I taken from bovine skin by Lai et al. (2008)^[^ ^105^ ^]^; Elastin taken from bovine ligament by Gosline and French (1979)^[^ ^106^ ^]^; Fibronectin taken from human plasma^[^ ^107^ ^]^ by Bhuvanesh et al. (2019); MAX1 gelated at pH 9 with borate and NaCl at 50°*C* by Micklitsch et al. (2015)^[^ ^108^ ^]^; RADA16‐I gelated at pH 7 with water at 20°*C* by Wang et al. (2017)^[^ ^109^ ^]^; Fmoc‐FF (amplitude sweep) gelated at pH 6 with water, NaOH and HCl at 25°*C* by Helen et al. (2011),^[^ ^88^ ^]^ (frequency sweep) gelated at pH 7 with water at 20°*C* by Smith et al. (2008)^[^ ^86^ ^]^; PA‐E3 gelated at pH 5 by CaCl_2_ and water at 25°*C* by Greenfield et al. (2014).^[^ ^46^ ^]^

This relationship is not mirrored when considering the hydrogel stiffness of pure gels of these materials that have been normalized by their concentration (see **Figure** **3**), where we see that the elastin gel is three orders of magnitude stiffer than the collagen gel. The importance of sample preparation and material source may once again play a role here: the elastin used by Gosline and French (1979) in this study was taken from whole pieces of bovine ligament that had other ECM components removed via autoclaving. Both collagen and elastin fibers in ligaments are longer than those found in other areas of the body, and are extremely aligned to optimally transfer forces between muscle and bone, which as we have seen from the law of mixtures increases the material's stiffness toward the upper end of the possible stiffness range. Additionally, mature elastin is highly covalently crosslinked via lysine oxidase enzymes, which result in regular allysine bonds along the fiber which stabilize the network and further increase its mechanical properties.^[^ ^62^ ^]^ In comparison, the collagen used by Lai et al. (2008) was taken from bovine skin, which in its native state is comprised of short, randomly oriented collagen fibers that are unconnected throughout most of their length.^[^ ^103^ ^]^ This was then ground in a mill to create a powder, a process likely to further shorten and detach its constituent fibers. The rheology results of elastin and collagen may therefore be seen as upper and lower bounds respectively for their possible range of behaviors.

In contrast, very little stiffness data exists for SSAPs or SSAPHs. The only data available for fiber stiffness is for Fmoc‐FF, which Helen et al. (2010) valued at 1.5 MPa.^[^ ^88^ ^]^ The hydrogel results listed in **Table** **2** have been calculated from the normalized rheology data displayed in Figure 3 using the equation *E* = 2*G*(1 − υ) (where υ is the Poisson's ratio, assumed to be 0.4, and *G* is the storage modulus of the sample at 1 Hz and <1% strain). This conversion is not fully accurate, as it assumes the material is both homogeneous, isotropic, and completely elastic —requirements which cannot be met by a multi‐component viscoelastic material. Nevertheless it is a good comparative tool. From this, Fmoc‐FF is shown to be the strongest SSAPH network at 870 Pa, followed by MAX1 at 650 Pa, PAE3 at 600 Pa, and RADA16‐I at 250 Pa.

**Table 2:** Mechanical Properties of SSAPs and ECM Components.

	Collagen‐I	Elastin	Fibronectin	MAX1	RADA16‐I	Fmoc‐FF	PAE3
**Monomer**							
MW (kDa)	300^[^ ^55^ ^]^	72^[^ ^38^ ^]^	500^[^ ^67^ ^]^	2.5^[^ ^110^ ^]^	1.9^[^ ^111^ ^]^	0.5^[^ ^112^ ^]^	1.2^[^ ^46^ ^]^
ϵ_*f*_ (%)	50^[^ ^58^ ^]^	800^[^ ^38^ ^]^	600^[^ ^113^ ^]^				
**Fibril**							
Diameter (nm)	40^[^ ^94^ ^]^	4^[^ ^65^ ^]^	5‐12^[^ ^39^, ^40^ ^]^	3.5^[^ ^108^, ^114^ ^]^	6^[^ ^83^ ^]^	3^[^ ^115^ ^]^	7^[^ ^116^ ^]^
**fiber**							
Diameter (nm)	800–10 000^[^ ^58^, ^95^ ^]^	100–200^[^ ^65^ ^]^	10–1000^[^ ^69^ ^]^	3.5^[^ ^108^, ^114^ ^]^	20–40^[^ ^78^, ^80^ ^]^	10–50^[^ ^45^ ^]^	20–40^[^ ^46^ ^]^
ϵ_*f*_ (%)	37^[^ ^58^ ^]^	200^[^ ^101^ ^]^	500^[^ ^69^ ^]^				
*E* (MPa)	3,520 ±6,600^a)^	1.2^[^ ^101^ ^]^	0.15 ±0.06^a)^		1.5±0.4^[^ ^88^ ^]^		
**Hydrogel**							
ϵ_*f*_ (%)				40–70^[^ ^73^, ^117^ ^]^	3^[^ ^109^ ^]^	0.02^[^ ^88^ ^]^	1^[^ ^46^ ^]^
*E* (kPa)	2.82±1.8^a)^	0.286±0.290^a)^	0.001^a)^	0.650^[^ ^108^ ^b)^	0.250^[^ ^109^ ^b)^	0.870^[^ ^88^ ^b)^	0.600^[^ ^46^ ^b)^

^a)^ Values taken from Table 1. Hydrogel values normalized by concentration.

^b)^ Values calculated using *E* = 2*G*(1 − υ), where υ is the Poisson's ratio, assumed to be 0.4, and from referenced rheology *G*′ values taken at 1 Hz, >1% strain, normalized by concentration.

ϵ_*f*_ = fracture strain.

Collagen and elastin may therefore be considered as the stronger ECM components, with fibronectin as a much softer material. All the studied SSAPHs are significantly stronger than fibronectin, and significantly weaker than collagen. Notably, the stiffness characteristics of RADA16 align it closely to elastin — the Young's modulus of the elastin fiber and the RADA16 fiber are 1.2 and 1.5 MPa respectively, alongside similar normalized hydrogel stiffnesses (250 Pa vs 286 Pa, respectively).

### 5.2 Viscoelasticity

ECM components are inherently viscoelastic.^[^ ^53^ ^]^ Low forces exerted over extended timescales result in the networks acting as a viscous fluid, while when exposed to higher forces over shorter timescales, they act as a series of simple connected springs. The Maxwell model conceptualizes this as a network made up of a dashpot and spring attached as a series circuit, describing the viscous and the elastic component respectively.^[^ ^118^ ^]^ The effect is a network which is time‐, force‐, and component‐dependent. In a SSAP network, the forces binding monomers together are significantly lower than those in covalently bonded structures. This makes it easier for these monomers to slide and relocate within their fibril, resulting in the prevalence of viscoelastic behavior in both ECM components and SSAPs due to inherent intrafibril viscous potential. An excellent minireview on the topic may be found in Elosegui–Artola (2021).^[^ ^119^ ^]^

The viscosity of a material can be ascertained from its loss modulus, i.e. the amount of energy lost to non‐elastic forces such as friction and heating. This can be divided by the storage modulus to produce a proportional viscosity value known as the *tan*(δ) that is independent of sample shape or size. Figure 3b,d show the values of *tan*(δ) for ECM networks and SSAPHs over different strains and frequencies.

The viscoelastic profile of the ECM components is distinct for each material. The viscous component of collagen I is seen to be largest for all the SAPs, and increases at lower shear up to 0.7 at 0.01 Hz. Unsurprisingly, the viscous component of Elastin is extremely low, but unlike collagen this value rises slightly with increasing oscillatory frequency from 0.01 to 0.05. Fibronectin lies between elastin and collagen with a viscous component of 20%, though this increases rapidly so that when under 10% strain 50% of this energy is lost via viscous mechanisms.

In contrast, the SSAPHs display highly similar viscoelastic profiles. The outlier is MAX1, whose low viscous component lies well below 0.1 until gel breakage. PAE3 and RADA16 have very consistent viscoelastic profiles, with both viscous components ranging between 0.2 and 0.3. Surprisingly, Fmoc‐FF's viscoelastic component increases with strain from 0.25 to 0.5 until gel breakage, opposing the trend seen in ECM components.

The viscous component of collagen is significantly higher than any of the SSAPHs. However, the viscoelastic profile of PAE3 overlaps considerably with that of fibronectin over 0.1–10% strain, with RADA16 also displaying very similar properties over 10–1000 Hz. In contrast, MAX1's high elasticity is far more akin to elastin, with a viscous component of under 0.1.

### 5.3 Extensibility

The breaking strain of monomers, fibers and hydrogels are displayed in Table 2. The elastin monomer is seen to have the greatest extensibility, able to elongate up to 8× its original length. Fibronectin monomers can stretch up to 6× before breakage, while tropocollagen can only extend by around 50% of its original length. Interestingly, this relationship does not hold at the fiber level, where fibronectin loses only a small fraction of its monomer's capabilities and is still able to extend by up to 500% in fiber form, whereas elastin is only able to extend by 200% as a fiber, a loss of 3/4. Collagen's extensibility also decreases to 37%, while under compression the fibers begin to buckle at only 5% strain.^[^ ^58^ ^]^ Collagen is therefore strong, but relatively brittle. In contrast, fibronectin fibers are twice as extensible as elastin fibers, and 10× as extensible as collagen.

The breaking strain of a material is when the relationship between strain and the material storage modulus is no longer linear. These values are listed in Table 2. The yield strain is where the *tan*(δ) becomes greater than 1 on an amplitude sweep, as this marks where the gel becomes a viscous fluid. The author was unable to find breaking strain values for ECM component gels in the literature, but in Figure 3 the yield strength can be seen to be reached by Fmoc‐FF at 1%, MAX1 at 30% and PAE3 at 35%. MAX1 possesses one of the highest breaking strains of all the SSAPHs, at 40%‐70%. However, this is comparatively small when compared to the extensibility of an elastin fiber. In contrast, the gel breaking strain for RADA16‐I is only 3%. Similarly Fmoc‐FF has the lowest breaking strain of all the SSAPHs, at only 0.02%, making it significantly more brittle than any of the ECM components and liquefying at only 5% strain (3.b)). PAE3 has a similarly low breaking strain, at only 1%. ECM components are therefore far more flexible than the studied SSAPH gels.

### 5.4 Shape

At the nanoscale, the size of molecules is most easily understood in terms of their molecular weight. Fibronectin is the largest monomer considered here at 500 kDa (considered in its dimer form), followed by tropocollagen at 300 kDa, and tropoelastin at 72 kDa. These monomers are much larger than the SSAPs', none of which have a size greater than 3 kDa. These differences will alter the kinetics of self assembly and may explain why small changes in factors such as pH and temperature result in such large differences between SSAP gels, particularly for the smaller molecular weight peptides such as Fmoc‐FF.

Fibril diameters, i.e., the most basic form of monomer chain, are fairly consistent across both ECM components and SSAPs. Collagen is an outlier, with a fibril diameter of 40 nm of regularly stacked tropocollagens that associate to create its crimped structure. The rest lie between 3 and 12 nm, with PAE3, fibronectin, and RADA16 on the larger end (7, 6, and 8.5 nm, respectively), and MAX1, Fmoc‐FF and elastin on the smaller (3.5, 3, and 4 nm respectively). This similarity is lost once the fibrils begin to associate to form fibers. The largest of these is collagen, with diameters from 800 nm, reaching up to 10 µm. Fibronectin fibers can remain at fibril size or associate up to 1 µm, while elastin has a fairly consistent range of 100–200 nm. In contrast, RADA16‐I, and PAE3 both have fiber ranges of between 20 and 40 nm. Fmoc‐FF has a slightly broader range of between 10 and 50 nm, while MAX1 is unable to form associations between its fibrils, resulting in a complete network consisting of only 3.5 nm fibrils. SSAPs are therefore comprised of smaller units, and thinner fibers than ECM components.

## 6 SSAP Epitope Addition

Biological epitopes are peptide sequences with bioactive properties. They are most commonly used in biomaterials as an adhesion ligand to encourage cell attachment to a bioinert matrix. Often these are taken directly from bioactive sequences within ECM components, for example, the widely used RGD peptide sequence is taken from the fibronectin domain FNIII.^[^ ^17^ ^]^ Depending on the epitope chosen the addition may also play a much greater role in cell response; various sequences can mimic growth factors or bind to them, trigger cell growth, matrix turnover, or even control the immunogenic response.^[^ ^17^ ^]^ Thus, the addition of a biological epitope is often a simple and effective way to increase cell attachment and proliferation in a given matrix. A comprehensive library of these sequences and their effects may be found by Ligorio and Mata (2023).^[^ ^17^ ^]^

It is often assumed in cell studies that the effects of ligand addition to SSAPs are due to the ligand binding alone. However, due to the characteristic small size of SSAPs, the addition of even a short epitope to a monomer can often have a profound impact on the structural dynamics of the resulting network. First, care must be taken that the addition of an epitope does not sterically interfere or alter the SSAP's self‐assembly mechanisms. Second, due to the high packing density of functionalized monomers per unit of space, an optimal mix between ligand‐presenting and non‐ligand‐presenting peptides must be found to allow the cell receptors enough space to bind.^[^ ^120^ ^]^ In most cases, interaction of adjacent epitopes is unavoidable, altering the mechanical properties of the network which in turn affects cell response to the system.

Few studies exist on how epitope addition to SSAPHs affect their mechanical properties, and refer principally to rheological data. The addition of cell‐adhesive sequences IKVAV, RGD and YIGSR by Sun et al. (2017) to RADA16‐I resulted in storage modulus values of 200, 40, 5 Pa respectively, while the addition of VAPG, RGDS and YICSR epitopes to the PA CH_3_(CH_2_)_14_CONH‐GTAGLIGQ by Anderson et al. (2009) produced elastic moduli at 1 Hz of 175, 100, and 50 Pa, respectively.^[^ ^121^ ^]^ Fmoc‐FF gels with epitope additions have been found to have different compatibilities with different cell lines.^[^ ^89^ ^]^ Du et al. (2020) combined three “biological motifs” to Fmoc peptides to mimic fibronectin (Fmoc‐GFFRGD), collagen (Fmoc‐GFFGER) and laminin (Fmoc‐DDIKAV). Fmoc‐GFFRGD combined with Fmoc‐GFFGER, and all three combined, formed gels with extremely high viscous components compared to their constituents' individual gels (G′,G″ of 10 Pa, 8 Pa and 15 Pa, 9 Pa, respectively), while Fmoc‐GFFGER with Fmoc‐DDIKVAV and Fmoc‐GFFRGD with Fmoc‐DDIKVAV formed stronger, less viscous gels (G′, G″ of 80 000 Pa, 200 Pa and 1000 Pa, 100 Pa, respectively). Incredibly, the strain‐failure of these gels ranged between 1.5% and 100%.

The range of storage moduli values due to epitope addition potentially allows secondary control of SSAPH mechanical properties via choice of additional ligand. Sun and Zhao (2012) were able to produce a 3× increase in the storage modulus of RADA16‐I by the addition of spider silk motifs GGAGS and GPGGY to each monomer, raising the storage modulus from 4000 to 11 000 Pa.^[^ ^122^ ^]^ Potentially, ELP sequences such as VPGXG (where X is a non‐proline amino acid)^[^ ^123^ ^]^ could be added in order to increase the fracture strain of an SSAP gel. However, added complexities such as the ratio of ligand‐presenting to non‐ligand‐presenting peptides and the co‐assembly of multiple peptides presenting different ligands may make these applications difficult to control.

While a vast range of mechanical properties may be achieved via monomer sequence, epitope addition, and gelation procedure, far fewer methods are available post‐gelation to control or edit the stiffnesses of SSAPHs. One interesting modification that can be applied to MAX1 is the inclusion of a tyrosine to the peptide chain, allowing the stiffness of the gel to be reliably tuned post‐network formation via the addition of Frémy's salt ((KSo_3_)_2_NO), which oxidizes the tyrosine and allows it to covalently bond with lysine. This irreversible reaction is concentration‐dependent, allowing the gel stiffness to be increased by up to 7× the initial modulus. The salt may then be washed out with no effect on cell viability.^[^ ^44^ ^]^ Another novel technique was discovered by Zhang et al. (2010), who found that PA‐E3 (C_16_V_3_A_3_E_3_(CO_2_H)) elevated to 80 °C for 30 min and allowed to cool formed a highly viscous solution. Upon addition of CaCl_2_, it produced a network of 4× greater stiffness than standard PA‐E3. The effect was due to layers of multiple stacked PA bi‐layers that had formed at the higher temperatures, which when cooled separated into large aligned bundles of fibers of around 40 nm diameter, significantly larger than the standard fiber diameters of 8 nm.^[^ ^124^ ^]^

## 7 Multi‐Component Hydrogels

This review has focused on the mechanical properties of ECM components in isolation, which has allowed us to highlight the heterogeneity present between different ECM components in the body and compare it to those of SSAPs. However, these structures naturally exist in tissue as a highly inter‐correlated network, and should be considered as such. Equally, research into SSAPs is most commonly done using mono‐component gels, but researchers in this area are increasingly interested in producing more complex systems with multiple components to more closely mimic the diversity seen in the natural ECM.^[^ ^125^, ^126^ ^]^ This section details the existing literature on combining ECM components, as well as how combinations of SSAPHs, and ECM with SSAPHs, affect gel formation and cell response.

### 7.1 ECM Multi‐Component Hydrogels

Decellularized ECM (dECM) is a common method of forming multi‐component ECM‐based hydrogels,^[^ ^93^, ^127^ ^]^ as it is organ‐specific and enables much of the original proteinous ECM composition to be preserved. Typically, porcine ECM from the organ of interest is decellularized and ground into a powder, before being dissolved in a protease solution to form a homogeneous pre‐gel.^[^ ^127^ ^]^ The gelation process is then triggered via temperature increase by direct injection into the patient.^[^ ^93^ ^]^ An excellent review by Saldin et al. (2017) describes the mechanical properties of the resulting gels between different organs. G' stiffness values range between 6 and 800 Pa for most organs, with only cartilage‐sourced gels reaching into the kPa range (4 kPa). Average fiber diameters are around 100 nm, but differ widely depending on species and ECM concentration used.^[^ ^93^ ^]^ While dECM gels are in clinical use due to their excellent biocompatibility and near 100% cell viability, clear issues exist with batch‐to‐batch variation (for example, human‐sourced dECM has difficulty reliably forming gels in vivo^[^ ^93^ ^]^), and the weak gels that form are at least an order of magnitude lower than the tissue they seek to emulate.^[^ ^2^ ^]^ For example, van Sprang et al. produced a dECM scaffold derived from porcine kidneys for comparison with a UPy‐HA+UPy‐PEG hybrid gel, and found that the dECM scaffold could reach stiffnesses only up to 0.2 kPa due to solubility limitations of the dECM powder,^[^ ^128^ ^]^ compared to the base stiffness of a porcine kidney of 40 MPa.^[^ ^2^ ^]^

Multi‐component ECM‐based hydrogels can also be produced by directly combining pure components, providing more control over the proportions of each. Research on gels combining collagen and hyaluronic acid are most common,^[^ ^129^, ^130^, ^131^ ^]^ followed by elastin and collagen gel mixes.^[^ ^131^, ^132^ ^]^ Fibronectin blends are less well researched, with only fibronectin and collagen,^[^ ^131^ ^]^ and fibronectin and hyaluronic acid^[^ ^133^ ^]^ found in the literature. Researchers seeking to combine elastin with hyaluronic acid typically utilize the SSAP ELP instead,^[^ ^134^, ^135^ ^]^ which is modeled from a repeating motif in the elastin monomer, and does not suffer from elastin's solubility issues. Studies involving gels formed of more than two ECM proteins are rare, and most of the studies listed above lack mechanical property data. Stiffness data taken from rheology results suggest similarly low gel stiffnesses of multi‐ECM gels which is comparable to that of dECM gels.^[^ ^131^, ^132^ ^]^ Multi‐ECM gel stiffness appears to be predominantly collagen‐mediated, as the addition of fibronectin and HA, and elastin, was measured by Diester et al. (2012) and Vazquez‐Portalatin et al. (2020) respectively and found no effect on gel stiffness.^[^ ^131^, ^132^ ^]^

To combat the low stiffness displayed by ECM‐component gels, crosslinkers or natural polymers (such as gelatin or alginate) are commonly combined to improve the gel stiffness and/or handling ability.^[^ ^136^, ^137^, ^138^ ^]^ One novel bottom‐up approach is to use a base scaffold to encourage cell ECM deposition, followed by decellularization, to maximize natural ECM structural formation in the final product. Shi et al. (2024) cultivated chondrocytes on micro‐porous GelMa scaffolds for 28 days to produce a GelMa‐ECM scaffold of 8 kPa stiffness, tangent modulus of 0.39 and a >90% breaking strain. This method, while effective, is resource intensive, and does not possess the injectable properties common to dECM gels and most SSAPs.

### 7.2 SSAPH Multi‐Component Hydrogels

Combining multiple SSAPs to form multi‐SSAP hydrogels is a very new research area. In theory, this method mimics the ECM microstructure far more closely than single‐SSAP gels, while achieving the mechanical properties multi‐ECM gels struggle to reach. The majority of studies in this field typically combine a base peptide structure with different bioactive epitopes to produce multiple SSAPs, which are combined and subsequently assemble into a scaffold with these bioactive ligands available to improve cell response. This method has been used to good effect with both PAs^[^ ^24^, ^139^ ^]^ and Fmoc gels.^[^ ^140^ ^]^ However, due to the sharing of gelation mechanism between the different peptides, the resulting scaffolds are comprised of a single homogeneous network, instead of the multiple discrete fibrous components which would be more akin to natural ECM.^[^ ^24^, ^141^ ^]^ Okesola et al. have done several studies using multiple gel components, with crucially different gelation mechanisms, producing a matrix of multiple fiber types.^[^ ^116^, ^142^ ^]^ Their 2019 study combines the charge‐based assembling PA‐E3 with a sugar‐based, low‐molecular‐weight gelator DBS‐COOH which assembles via π−π stacking and hydrogen bonding. The resulting gel stiffness increased linearly with DBS‐COOH content from 9 kPa (E3‐alone) to 20 kPa (6:4 ratio PA:DBS‐COOH) to 27 kPa (DBS‐COOH), with good cell response.^[^ ^116^ ^]^

### 7.3 ECM‐SSAPH Multi‐Component Hydrogels

SSAPs with ECM components seeks to combine the bioactivity of the natural ECM with mechanical properties of SSAPHs. While this is another very new area of research, of all ECM and SSAPH combinations, this method has shown particularly reliable success in‐vivo. Better spinal fusion in rats and rabbits was found by Weiner et al. (2016) when using a collagen and PA‐E3 gel compared to a collagen‐alone control,^[^ ^143^ ^]^ and Osuna de la Peña et al. (2021) found that while PAs combined only with collagen formed thick fibers, when combined with collagen, fibronectin, laminin, and hyaluronic acid they produced a fine fibrous meshwork of 1 kPa, which performed better than matrigel at reproducing the in‐vivo cell response of PDAC.^[^ ^144^ ^]^ Okesola et al. (2020) combined a PA with hyaluronic acid functionalized with tyramine, alongside laponite (a synthetic clay) for bone regeneration. This produced a fibrillar nanostructure of 60 kPa gel, with stress relaxation profiles to match those of bone. The gel performed better than Bio‐Oss in an in‐vivo bone regeneration study, and promoted osteogenic differentiation and angiogenesis in hUVECs in vitro without the use of growth factors.^[^ ^142^ ^]^ These successes display the great promise shown by multi‐network SSAP‐ECM hydrogels to provide bioactivity with tissue mechanical matching. Developing similar studies using Fmocs, β‐sheets and β‐hairpin peptides combined with ECM components would be welcome to broaden the range of SSAPHs involved.

## 8 Mechanotransduction and Cell Response

Mechanotransduction is the transferal of mechanical information between the cell and its environment into biochemical signals, which in turn affect intracellular signaling and DNA transcription.^[^ ^145^, ^146^ ^]^ This process results in a feedback loop where ECM production and degradation is directed by the mechanical properties of existing ECM, and which regulates fundamental cell properties such as cell fate, propagation rate, migration direction and even apoptosis. For those working in biomaterials, this raises the fundamental problem ‐ in such a complex, interconnected system, how can we create the perfect environment for cells, without the cells? To answer this, investigation into the effect and significance of individual mechanical factors is a pivotal research area, whose broad findings are covered in this section.

Mechanotransduction between the cell and the ECM typically occur through integrin‐mediated pathways, where integrins on the cell membrane bind directly to binding sites on ECM proteins such as collagen I.^[^ ^145^ ^]^ Many ECM networks exist in a state of constant tension via this mechanism,^[^ ^69^, ^147^ ^]^ which opens up binding sites within their constituent monomers, resulting in cell‐signaling cascades that affect cell processes.^[^ ^148^ ^]^ This tension also directly changes the internal mechanics of the cell cytoskeleton: in response to connections to stiff ECM components, integrins cluster into focal adhesion points around which scaffolding proteins assemble.^[^ ^50^, ^149^ ^]^ These proteins trigger the formation of a network of thick cable‐like actin stress fibers in the cytoplasm. Cell glycolysis is believed to be regulated in part by mechanical cues from these stress‐fiber networks: Park et al. (2020) discovered that when actin filaments in human bronchial epithelial cells are bundled and placed under tension, binding sites open up which allow proteins such as the degradation‐labeling enzyme TRIM21 to attach.^[^ ^150^ ^]^ When TRIM21 is inactivated by this process, metabolic pathways are triggered that increase cell glycolysis.^[^ ^50^ ^]^ Cells in a stiff ECM network therefore have a faster metabolism than those in a soft ECM.^[^ ^50^ ^]^

Cell migration speed is also partially dependent on substrate stiffness. On surfaces with greater ligand availability, maximum migration speed of multiple cell types including DU‐145 carcinoma cells occurs when the material is soft, while when ligand availability is low the cell's maximum migration speed is reached only via a stiffer substrate,^[^ ^149^ ^]^ suggesting an almost trampolining effect where the cell uses the elastic energy of the substrate to drive its motion forward.

The use of integrin binding sites and their ability to form focal adhesion points enables cells to sense and respond to the precise stiffness of their environments,^[^ ^151^ ^]^ allowing substrate stiffness to be a direct programmer of cell fate.^[^ ^145^, ^152^, ^153^ ^]^ AFM nanoindentation on thin polyacrylamide gels by Engler et al. (2006) found that MSCs grown on gels with Young's modulus between 0–2 kPa displayed neuronal differentiation markers, while those on 2–20 kPa gels displayed myogenic differentiation markers, and those on 15–70 kPa gels displayed osteogenic differentiation markers.^[^ ^154^ ^]^ Similarly, compression testing of 3D alginate and agarose gels by Huebsch et al. (2010) which had been functionalized with integrin‐binding peptides and photo‐crosslinked found osteogenesis of hMSCs to occur on gels between 11 and 30 kPa stiffness, and adipogenesis between 2.5 and 5 kPa.^[^ ^155^ ^]^ This effect is found in even embryonic stem cells: Zoldan et al. (2011) used tensile testing of synthetic polymer scaffold (PEGDA or PLLA) and found that while hESC did not differentiate above 6,000 kPa substrate stiffness, mesoderm differentiation occurred between gels of 6,000‐1,600 kPa, endoderm differentiation between 100 and 1000 kPa, and ectoderm differentiation below 100 kPa gel stiffness.^[^ ^3^ ^]^ This display of clear stiffness boundaries between differentiation pathways across multiple material types is a fascinating insight in a field which predominantly uses tissue culture plastic (3 GPa^[^ ^156^ ^]^) and glass (70 GPa^[^ ^157^ ^]^) during cell growth and passaging.

However, stiffness is not the only factor in mechanotransduction. In a viscoelastic environment the stored energy from cell‐ECM interactions is dissipated by viscous mechanisms or plastic deformation over time.^[^ ^53^ ^]^ There is some interest that cells could communicate via elastic wave packets propagating through the ECM matrix, which would reduce the impact of viscoelastic damping effects and pass information over greater distances, but this is an exploratory area that has not passed the modeling stage.^[^ ^158^ ^]^ However, it is known that the dissipation energy of a network directly impacts cell adhesion; Sacco et al., 2020 tested mouse fibroblast‐like and human osteosarcoma cells in 2D on different chitosan‐TPP/PPi substrates and found that low dissipation energy (derived from rheological measurements of strain‐softening) of 0.19 ± 0.09 J mol^−1^ resulted in high cellular adhesion and spreading. In contrast, moderate dissipation energy (0.42 ± 0.29 J mol^−1^) resulted in adhesion but reduced spreading, and high dissipation energy (1.58 ± 0.47 J mol^−1^) resulted in limited adhesion and spreading.^[^ ^53^ ^]^

Cells have been also found to take instruction from dynamic processes such as stress‐relaxation and stress‐stiffening, with faster relaxation times and later stress‐stiffening associated with bone‐like matrix production in MSCs.^[^ ^159^, ^160^ ^]^ Cells are believed to sense these effects predominantly via ion‐channel mediated mechanotransduction pathways, which are triggered via cell volume expansion and act to sense confinement.^[^ ^119^, ^145^ ^]^ Indana et al. (2022) modulated viscoelasticity and stress relaxation rate by changing the molecular weight of RGD‐conjugated alginate gels, and found that in 3D environments, a tangent modulus of 0.08–0.09 was found to increase cell cluster size in hiPSCs, while a tangent modulus of 0.06 triggered apoptosis at low RGD densities.^[^ ^161^ ^]^ Using the same materials, Lee et al. (2019) found that single‐cell volume expansion associated with enhanced osteogenic differentiation in MSCs occurred in their more viscoelastic alginate hydrogels, via TRPV4 ion channels.^[^ ^162^ ^]^

Another important mechanical factor is fiber diameters, which are also important in directing cell fate. A study by Abagnale et al. (2015) compared cell response to etched glass ridges coated with polyimide and found adipogenic differentiation of MSCs occurred on 15 µm‐diameter ridges, and osteogenic differentiation on 2 µm‐diameter ridges.^[^ ^163^ ^]^ Cartilage formation by bovine chondrocytes occurs to a greater extent on nanofibers (<400 nm) and larger microfibers (3–14 µm) than intermediate fibers.^[^ ^164^ ^]^ Cell stemness is also maintained better on PCL fibers, with 290 nm fibers providing a 3–4‐fold increases in stem marker expression of hMSCs compared to flat PCL surfaces.^[^ ^165^ ^]^ Multiple cell types including glioblastoma cells migrate faster on nanofibers (200–700 nm) compared to microfibers (1.1–5.7 µm) on gels of low stiffness.^[^ ^164^ ^]^

Another important factor is ECM loading, which influences the production of ECM proteins, as well as the turnover of these proteins via matrix metalloproteinases. Strain on collagen fibers is suggested to sterically prevent enzymatic degradation by increasing fibril packing density via loss of crimping.^[^ ^166^ ^]^ Further, loading of the ECM by cells modulates the stiffness of the local microenvironment: Han et al. (2018) used optical tweezers to discover that contractile forces by the cell on the ECM acts to locally stiffen the microenvironment in collagen, fibrin, and matrigel gels.^[^ ^167^ ^]^ Cells have also been found to form more stable focal adhesion sites when these sites are aligned with fiber direction, versus those bound perpendicular to their fiber length.^[^ ^168^ ^]^ This effect is believed to be due to differences in fiber stiffness experienced by the cell between these two states, and highlights the heterogeneity of cell experience even in an apparently homogeneous network.

Finally, fiber breaking strain is also associated with cell proliferation, and cell fate. Du et al. (2020) investigated the effect of the addition of epitopes to Fmoc peptides (Fmoc‐GFFRGD, Fmoc‐GFFGER, and Fmoc‐DDIKVAV) on the Fmoc gel mechanical properties. They discovered a positive correlation between the strain‐failure of the gels and cell proliferation, with a 2.5× increase in cell number when gel breaking strain was greater than 80%.^[^ ^141^ ^]^ Another example is that of Pek et al. (2009) used thixotropic 3D gels (PEG‐silica functionalized with RGD) and found that liquefaction stress was associated with MSCs differentiation markers ‐ neuronal markers peaked at 7 Pa, myogenic at 25 Pa and osteogenic at 75 Pa.^[^ ^169^ ^]^

These studies on mechanotransduction increasingly highlight the complexities involved both in cell‐ECM interactions, as well as the measurement of them. Experimental design is a challenge when factors such as stiffness, crosslinker density, porosity, and fiber diameter are so deeply interrelated, and these difficulties are compounded when cell response differs between setups (e.g., cells respond differently to 2D and 3D environments^[^ ^145^ ^]^). However, broad trends are becoming clearer on this subject, and can be used to guide mechanical considerations during the exploration of existing and development of novel biomaterials.

### 8.1 Applications to SSAPH Development

We have described in the previous section how growing cells on a soft matrix with small fiber diameters, low dissipation energy, and high breaking strain results in faster migration, slower differentiation, greater adhesion, and greater proliferation of these cells. These responses are all associated with increasing cell numbers throughout the matrix and facilitating new growth. Almost unsurprisingly, these factors are the properties associated with fibronectin, which is the first ECM component to assemble after injury. In contrast, a stiff matrix, with a low breaking strain and moderate dissipation energy results in cells that have a faster metabolism, limited spreading, faster differentiation and low proliferation. This is associated with an environment under regular load, such as bone or ligament, tissues which are predominantly comprised of collagen.

It is likely that cells are tuned to particular ECM components, not only via their chemical binding sites, but also via the mechanical factors explored in this review. Instead of mirroring the bulk properties of particular tissues, researchers into SSAPHs should be exploring ways to increase their SSAP's mechanical similarity to more closely mirror the microenvironment of these tissues, specifically modulating their SSAPs to emulate specific ECM proteins. This novel research direction would focus on developing hydrogels involving multiple SSAPs and/or ECM components, with differing gelation mechanisms and bioactive epitopes, each providing mechanical diversity for a different ECM‐component niche.

The SSAPs in this review may be reconsidered for their suitability for emulating specific ECM components. Broadly, the SSAPHs focused on in this review have nano‐sized diameters, moderately stiff matrices, low breaking strain and moderate viscoelasticity, except MAX1, which has a moderate breaking strain and high elasticity. Particularly, MAX1 exhibits elasticity close to that of elastin, and one of the highest breaking strains of the reviewed SSAPs. This is likely due to its β‐peptide monomer structure, which means that when its fiber is stretched each monomer can open its two folded halves like scissors, which then associate again once the force is removed.^[^ ^73^ ^]^ Modulation of MAX1 fiber stiffness via epitope addition at the “corners” of the folded peptide could increase the fiber stiffness via intrafibril crosslinking with minimal impact on this method of extensibility, and allow the SSAP to more closely approximate elastin. Similarly, RADA16 is an excellent candidate to mirror fibronectin due to its bioactive adhesion profile and similar viscoelastic response, and could be made weaker and more extensible to more closely resemble this ECM component. The ability of PAs to bundle after heat treatment could allow it to more closely mirror collagen, after modulation of PA stiffness via epitope addition. Finally, the versatility of Fmoc SSAPs we have seen in this review has suggested that this SSAP could easily produce a similar mechanical profile to any ECM protein. Here, due to the enormous range of mechanical properties seen with only minor alterations of epitope due to the SSAP's small size, the involvement of machine learning may be advantageous to predict mechanical changes and narrow down potential epitopes to more closely emulate the ECM component of choice.

## 9 Conclusion

In this review, we find that the mechanical properties of the ECM components collagen I, elastin, and fibronectin vary considerably: the Young's modulus of these materials ranges from kPa to GPa, maximum fiber extension differs significantly from 37% to 500%, and each component has a wide range of fiber diameters, with maximum fiber diameter 50–100× greater than their component fibrils'. The rheology data collated for these components also displays an impressive range of normalized storage moduli (10^−3^ − 10^6^ Pa/mg mL^−1^), each with a different dynamic relationship with its corresponding loss moduli. In contrast, the reviewed SSAPHs have maximum gel extensions of 1–100%, estimated Young's moduli within an order of magnitude of each other, and maximum fiber diameters of between 5 and 10 times their minimum fibril diameters. Their rheology results display much more stable behavior across shear frequencies and low strains, and have a smaller moduli range (10^0^ − 10^6^ Pa/mg mL^−1^). Each of these properties is known to have a direct impact on cell response via microscale interactions with their environment. The lack of Young's modulus values for SSAP fibers, and the significant range of values found for individual ECM components such as collagen, is a surprising hole in the literature and recommends greater research. A broad range of characterization methods for this purpose may be found in Bock et al. (2024)'s excellent review.^[^ ^170^ ^]^ Further properties such as the pore size, dissipation energy, Poisson's ratio, relaxation time and diffusivity of each of these materials are also important parameters when considering cell health and response, and would merit further study.

Currently, a mechanics‐oriented approach to SSAP‐based biomaterial design has focused on tuning a single hydrogel‐forming SSAP to match the bulk mechanical properties of the organ of interest, with the fixation of biological epitopes to create a suitable environment a cell can recognize and thrive in. This method often neglects the important component of microscale mechanics to cell health and direction, and presents an either‐or approach to ECM component gels. This review proposes that greater interest should be taken in modifying novel hydrogel components to more closely match the mechanical properties of specific ECM proteins, rather than tailoring toward the bulk organ. Additionally, the inclusion of natural ECM components in combination with one or several SSAPs would promote maximum bioactivity and micro‐mechanical control. This method would more closely align the cellular environment to the micro‐mechanics found in the natural ECM, with direct effects on intracellular processes, health, and differentiation. The tissue engineering applications of this method are extremely broad, as once the ratios of individual ECM components within a particular tissue are known, they can be mirrored via incorporation of their mimetic SSAPs to produce identical mechanical profiles, furthering research in areas from bone regeneration, to cancer cell models, to spinal cord repair.

## Conflict of Interest

The authors declare no conflict of interest.
